# Successful surgical management of a rare esophageal inflammatory myofibroblastic tumour: a case report

**DOI:** 10.1186/s13019-015-0327-5

**Published:** 2015-09-09

**Authors:** Prabhat Khakural, Ranjan Sapkota, Uttam K. Shrestha, Prakash Sayami

**Affiliations:** Department of CTVS, Manmohan Cardiothoracic Vascular and Transplant Centre, Kathmandu, Nepal

**Keywords:** Dysphagia, Inflammatory myofibroblastic tumour, Submucosal mass

## Abstract

Inflammatory myofibroblastic tumour (IMT) is an uncommon mesenchymal tumour, which can occur anywhere in the body, rarely in esophagus. Mostly, the diagnosis is postoperative, after the hispathological evaluation of the specimen. There are no definite guidelines regarding the diagnosis and management. Here, we report a 60 year old lady presenting with dysphagia, diagnosed to have a submucosal esophageal tumor with Barium esophagogram and contrast enhanced computed tomography. She was managed successfully with surgical enucleation with the final histopathological diagnosis of IMT. Surgical excision is not only therapeutic but also diagnostic in such cases.

## Background

Inflammatory myofibroblastic tumor (also known as plasma cell granuloma) is a rare mesenchymal tumor. It is a distinctive lesion composed of myofibroblastic spindle cells with an inflammatory infiltrate of plasma cells, lymphocytes, and eosinophils. It occurs most commonly in the lungs and uncommonly in the sites such as brain, trachea, breast, spleen, kidneys, liver, stomach and the ampulla of Vater. It is extremely rare in the esophagus and there are very few cases of esophageal IMT reported in English literature till date. It is seen mostly in children and young adults but can occur in any age, affecting females and males equally (female to male-1:1.4) [1].

We report an elderly female who underwent complete excision of the esophageal IMT and is doing fine without evidences of recurrence at follow up visits.

## Case presentation

**Fig. 1:**
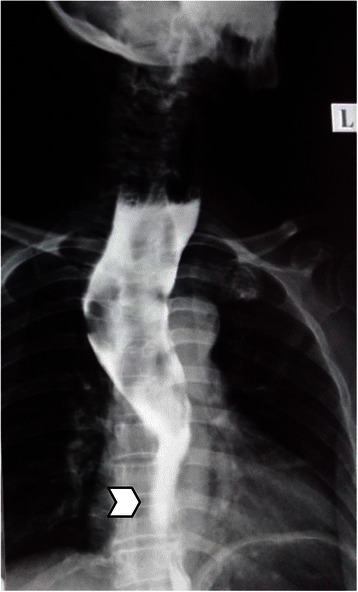
Barium swallow: arrow head showing smooth narrowing of the mid esophagus

**Fig. 2:**
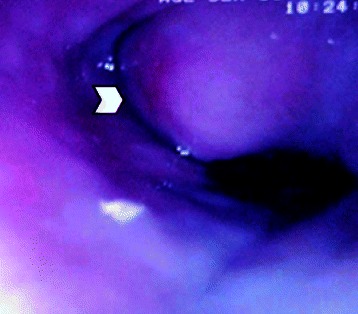
Esophagoscopic picture: arrow head showing the esophageal submucosal growth with an intact mucosa

**Fig. 3:**
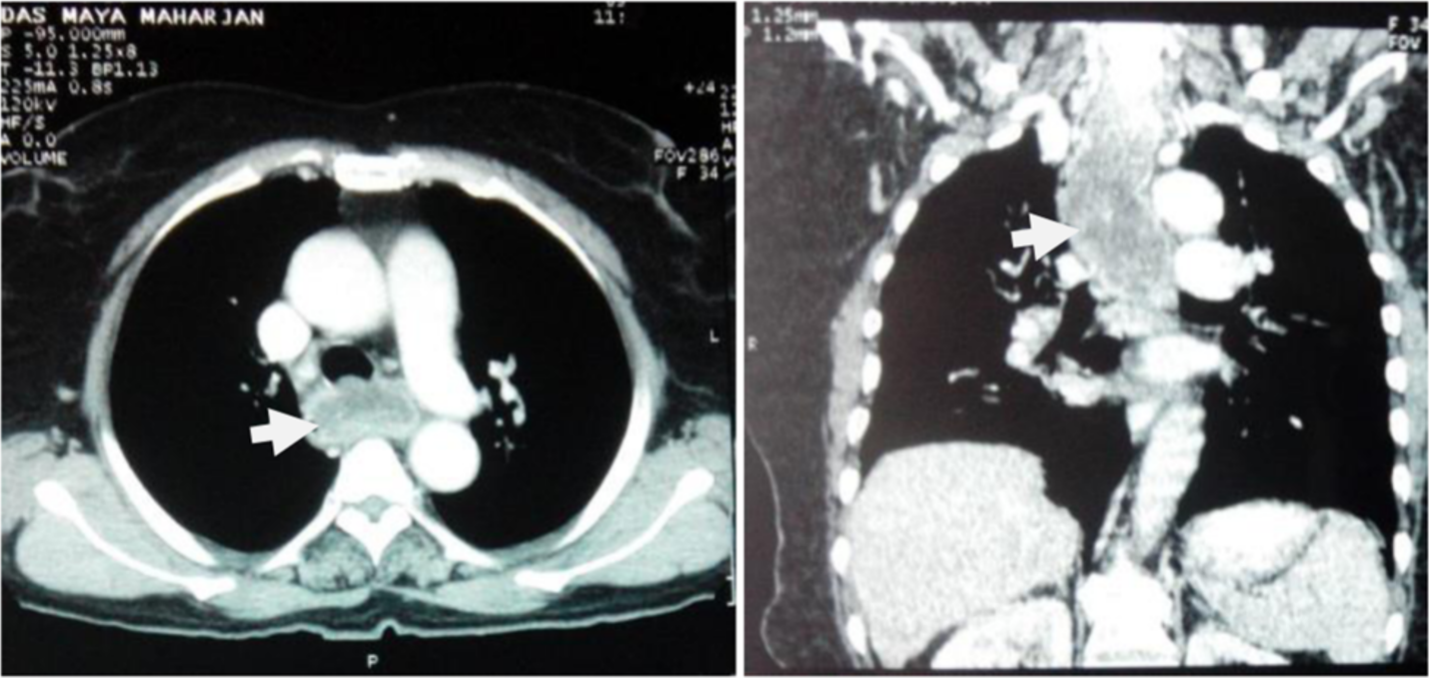
CT images: arrow showing the esophageal mass

**Fig. 4:**
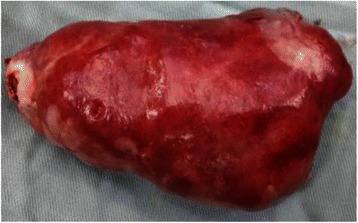
Esophageal mass

**Fig. 5:**
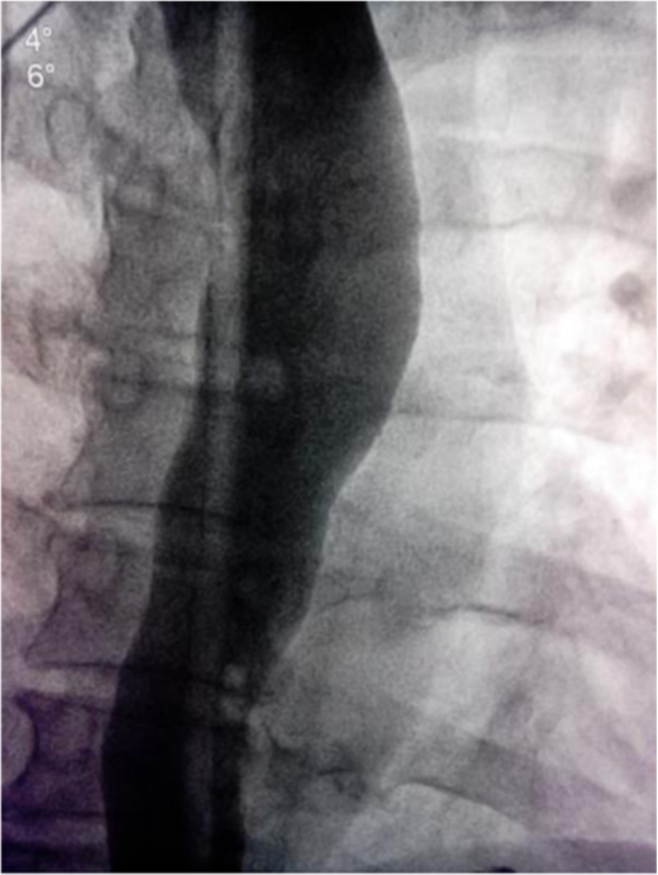
Postoperative gastrograffin swallow: normal caliber esophagus with no contrast extravasation

**Fig. 6:**
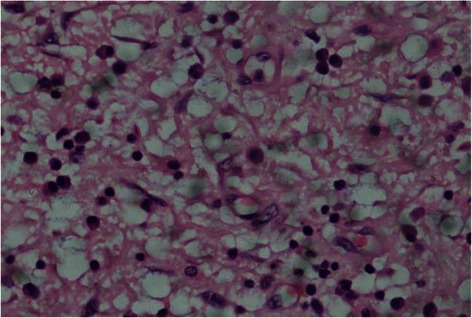
Microscopic study: slide showing plasmacytic infiltration

A 60 year old diabetic, non smoker, housewife presented to us with complaints of gradual onset, progressive dysphagia initially for solid and later for liquid food for one and a half year and foreign body sensation in throat for 6 months. She had lost 10 kg over last 6 months, despite having a good appetite. She did not have significant medical or surgical illness or intervention in the past. General and systemic clinical examination was normal. Her hematological and biochemical investigations were normal too. Barium esophagogram revealed smooth narrowing of the mid thoracic esophagus with proximal dilatation (Fig. 1). Esophagoscopy was suggestive of a submucosal growth with intact mucosa from 18 to 25 cm from central incisors, involving half of the circumference of esophagus, located at 12 to 6 o’ clock position (Fig. 2). Contrast enhanced computed tomography (CECT) scan of neck, chest and abdomen revealed a longitudinally oriented, well defined, non-enhancing, homogenous lesion involving the lower cervical and upper thoracic esophagus, causing significant luminal narrowing (Fig. 3). The patient underwent a right lateral thoracotomy. Esophageal dissection was done and longitudinal incision was made in the esophagus, over the lesion which was deepened through the muscle layer and enucleation of the lesion was performed. It was a solid, submucosal mass measuring 8.5 × 6 × 2.5 cm (Fig. 4). While dissecting the mass out, there was a 6 cm long clear rent in the mucosa but as the tissue was healthy, uninflamed and well vascularised, it was primarily repaired in two layers and reinforced with adjacent pleural flaps. She was kept on partial parenteral nutrition, intravenous Pantoprazole, nasogastric drainage and enteral feeding was done through feeding jejunostomy. Contrast esophagogram on seventh postoperative day revealed normal esophagus (Fig. 5). She was started on liquid diet initially and later she could swallow both solid and liquid food without dysphagia. She was discharged from hospital on tenth postoperative day. Gross pathology demonstrated an encapsulated solid mass with cut surface showing solid white areas without hemorrhage, necrosis and calcification. The microscopic examination of the mass revealed proliferation of spindle cells with stroma of proliferative blood vessels and lymphoplasmacytic infiltration with formation of lymphoid follicles (Fig. 6). The cells were immunonegative for Anaplastic Lymphoma Kinase (ALK). The patient is on regular follow up. At 6 months postoperatively she is doing well, without recurrence of her symptoms. Esophagoscopy done revealed normal esophagus.

## Discussion

The etiology of IMTs is supposed to be an aberrant response to tissue injury with myofibroblast being the primary cell type [2]. The commonly reported etiologies of IMTs include Epstein Barr virus, human herpes virus eight infection, trauma, reflux and overexpression of interleukin 6 [3, 4]. IMT, although once thought to be benign, is now considered to be an intermediate-grade tumor with potential for recurrence [5]. The most frequent symptoms of IMTs of the esophagus are dysphagia (as in our patient), substernal pain and inflammatory features related to cytokine release like fever, weight loss and raised acute phase reactants. Interestingly, the latter features were absent in our patient. Endoscopic Ultrasound (EUS) is the diagnostic test of choice, which can show hypoechoic lesion of the muscularis propria. But EUS characterization and final pathology are correlated in only 77 % of cases [6]. However, other investigation modalities can be of substantial help in diagnosing the condition. A chest X-ray can demonstrate a peri-hilar shadow if the tumour is big enough. Barium swallow may demonstrate smooth tapering and an even filling defect in the esophagus [7]. Esophagoscopy usually shows a submucosal growth similar to that of leiomyoma. CECT scan demonstrates a homogenous soft tissue lesion in the esophagus [8]. Our patient demonstrated all the features on corresponding investigations. The differential diagnoses of IMTs include other submucosal lesions like leiomyoma and gastrointestinal stromal tumours. Esophageal IMTs are found most commonly in the thoracic portion, followed by cervical and the distal esophagus [9]. These generally appear as small nodules or circumscribed masses and are frequently associated with mucosal ulceration. However the tumour can range from 1.5 to 20 cm in length [10]. Grossly, these are firm tumours composed of spindle shaped myofibroblastic cells with plasmacytic infiltration. The cells are immunopositive for vimentin, smooth muscle actin and immunonegative for CD34, CD117, and S100 [1]. More than 50 % of IMTs are reactive for ALK protein. Although ALK reactivity is not specific to IMT, it appears to be a factor associated with metastasis and recurrence [11]. Surgery is the treatment of choice, with esophagotomy and enucleation being the commonest procedure. However there are reports of such cases being managed by observation, medical management with proton pump inhibitors, esophagectomy, endoscopic resection and chemotherapy and radiation (in large, unresectable tumours). Prognosis is favourable without recurrence in the majority of the cases.

## Conclusion

Although esophageal IMT is an extremely rare entity, it should always be considered a differential diagnosis in a patient presenting with dysphagia and submucosal esophageal tumour. The complete excision of the tumour is not only therapeutic but also helps to confirm the diagnosis with histopathological examination and immunohistochemistry. As it is an intermediate grade tumour with recurrence potential, patient should be followed up regularly.

## Consent

Written informed consent was obtained from the patient for publication of this case report and any accompanying images. A copy of the written consent is available for review by the Editor-in-Chief of this journal.

## Abbreviations

IMT: Inflammatory myofibroblastic tumour
CECT: Contrast enhanced computed tomography
ALK: Anaplastic Lymphoma Kinase
EUS: Endoscopic ultrasound
